# Screening Methods for Antimicrobial Residues in the Dairy Chain—The Past and the Present

**DOI:** 10.3390/antibiotics13111098

**Published:** 2024-11-19

**Authors:** Pavlína Navrátilová, Lenka Vorlová, Sandra Dluhošová, Klára Bartáková, Oto Hanuš, Eva Samková

**Affiliations:** 1 Department of Animal Origin Food & Gastronomic Sciences, Faculty of Veterinary Hygiene and Ecology, University of Veterinary Sciences Brno, 61242 Brno, Czech Republic; navratilovap@vfu.cz (P.N.); dluhosovas@vfu.cz (S.D.); 2 Dairy Research Institute Ltd., 16000 Prague, Czech Republic; hanus.oto@seznam.cz; 3 Faculty of Agriculture and Technology, University of South Bohemia in České Budějovice, 37005 České Budějovice, Czech Republic; samkova@fzt.jcu.cz

**Keywords:** residues, antibiotics, analysis, milk, milk safety

## Abstract

The presence of residues of antimicrobial substances in milk has been an important hygienic and technological parameter of raw milk quality since the 1960s. The presented review focuses on screening methods (microbiological inhibition methods and rapid specific tests) that are used in the control of antimicrobial residues in milk in the context of their historical development up to the present. We briefly explain the principles of the methods and discuss their pros and cons. The aim was to provide both the historical perspective on this topic and provide useful information on screening methods that are currently routinely used for the detection of residues of antimicrobials at farms, in the dairy industry, and in milk quality control laboratories.

## 1. Introduction

The use of antimicrobials in veterinary medicine started soon after these compounds were discovered. The discovery of penicillin (1929) was one of the most important milestones in this field. Penicillin was the first antibiotic globally used in the treatment of mastitis. The discovery of sulfonamides and their antimicrobial properties in 1932 and their introduction into medical and veterinary practice was another breakthrough in fighting bacterial infections. Many antimicrobials available between 1940 and 1965 are still used in veterinary medicine today. Nowadays, however, the range of veterinary medicines is wide. Tetracyclines, β-lactams, aminoglycosides, macrolides, and sulfonamides are the most widely used antimicrobials in food animals [1,2,3]. The main causes of antibiotics use in dairy farms are clinical mastitis, bovine respiratory disease, metritis, dry cow therapy, claw horn lameness, digital dermatitis. In dairy cows, antibiotics used for mastitis treatment include first-generation cephalosporins (cefazolin), second-generation cephalosporins (cefprozil), third-generation cefalosporins (ceftiofur), streptomycin, ampicillin, cloxacillin, and penicillin. For respiratory disorders in cattle, antibiotics like tilmicosin, florfenicol, enrofloxacin, and ceftiofur are used. Antibiotics used to treat foot infections include sulfonamides, β-lactams, tetracyclines, and lincomycin. Bacterial skin disorders in cattle are commonly treated with tetracyclines, β-lactams, macrolides, aminoglycosides, and fluoroquinolone [4].

From a historical perspective, the analysis of antimicrobial residues in food is a relatively young discipline. The requirement for regular monitoring of antimicrobial residues in milk started to be implemented only in the 1960s. For example, in the BENELUX countries, residue analysis started in the late 1960s and 1970s. In one of the federal states of former West Germany, milk from farms was regularly tested and paid for on the basis of a fermentation test prescribed by the legislation; since 1972, a Brilliant Black Reduction Test was introduced to control for the presence of residues [5,6].

The first concerns related to the presence of antibiotic residues in milk were raised by milk processors who noted that milk contaminated with antibiotics inhibited the growth of starter cultures used in the dairy industry and caused problems, in particular, in the production of fermented dairy products and cheeses. In the 1940s, therefore, developing and implementing sensitive and reliable methods for the determination of penicillin residues (and, later on, residues of other antimicrobials) became an urgent requirement of the dairy industry [7]. 

In the early 1960s, attention started to be paid to the risks posed by antibiotic residues in milk to consumers. This led to the first steps aiming to limit the presence of antibiotic residues in milk. The introduction of the first withdrawal periods for antimicrobial substances used to treat dairy cows was of great importance for the protection of consumer health and ensuring the technological safety of the raw material (raw milk). During this period the milk must be discarded. In 1951, the American Dairy Science Association passed a resolution stipulating that no milk intended for human consumption can be obtained from a mastitis-affected quarter for 72 h (six milkings) after the last application of the drug. In the United States, the Food and Drug Administration (FDA) concluded in 1955 that consumption of antibiotic residues-containing foods represents a health risk to consumers. Since then, the presence of antibiotic residues in food for human consumption has been considered milk adulteration [8].

As reported by Heeschen et al. [9], the issue of chemical residues in food began to be intensively addressed by the International Dairy Federation (IDF). The activities of the IDF in the period of 1968–1991 were summarized in the Special Issue 9101- Monograph on Residues and Contaminants in Milk and Milk Products [9].

Discussion on the potential risks associated with antibiotic residues in milk led to the establishment of the upper limits acceptable for specific compounds in milk. According to the Directive EEC 85/397 raw milk intended for the manufacturing of dairy products had to meet the following standards for antibiotic residues on receipt to the processing plant: “penicillin concentration below 0.004 µg/mL and undetectable residues of remaining antibiotics and sulphonamides”. In practice, these measures led to establishing analytical methods for penicillin detection with a limit of detection (LOD) of 4 µg/kg without paying detailed attention to other antibiotics [6,10,11].

The activities of Codex Alimentarius, a joint FAO/WHO program since 1985, played an instrumental role in establishing the first standards for residues in foods. These standards were based on a scientific assessment carried out by the Joint FAO Expert Committee on Food Additives, which recommended maximum residue limits (MRLs) for individual substances based on acceptable daily intake (ADI) values. The MRLs definitions were determined on the basis of the pharmacokinetic and toxicological properties of the individual compounds and their metabolites, and the ADI values based on the non-observed-effect levels or non-observed-adverse-effect levels [11,12,13]. In 1990, MRLs for residues of veterinary medicinal products in raw foodstuffs and foods of animal origin were established by EU legislation. The term “Maximum residue limit”, according to the Council Regulation (EEC) 2377/90 [14], is defined as “the maximum concentration of a residue resulting from the use of a veterinary medicinal product (expressed in mg/kg or μg/kg of fresh tissue) that can be recognized by the Community as legally permitted or acceptable in/on foods”. 

Besides MRLs, maximum concentration levels have been established for antimicrobial residues in milk according to the Codex Alimentarius (Codex maximum level). Codex maximum limit for residues of veterinary drugs (MRLVD) is the maximum concentration of residues resulting from the use of a veterinary drug (expressed in mg/kg or µg/kg on a fresh weight basis) that is recommended by the Codex Alimentarius Commission to be legally permitted or recognized as acceptable in or on a food [15,16]. 

The activities of the IDF focusing on methods for detecting antimicrobial residues in the milk chain were also important. In 1987, IDF published Bulletin No. 220—Detection of Inhibitors: Reference, Routine and Confirmation Methods. A later, second, edition of that Bulletin of IDF No. 258 was also dedicated to methods of detection of residues of inhibitory substances [17,18].

The analytical procedures published by the IDF in Bulletin No. 220/1987 [17] were used as a basis for procedures prescribed for the detection of antibiotics and sulfonamides in raw and heat-treated milk established in the Commission Decision No. 180/1991 [19]. In that Decision, the reference method for detecting antibiotic and sulfonamide residues in raw and heat-treated milk (Chapter VIII.) included a qualitative method using *Bacillus stearothermophilus* var. *calidolactis* ATTC 10149 as the test micro-organism and a method for the confirmation and identification of penicillin. 

A subsequent IDF monograph aimed to identify the main routes of entry of residues and contaminants into milk and to determine appropriate remedial measures [20]. The document described the principles of the HACCP (Hazard Analysis and Critical Control Points) system (concept and its application), the principles of toxicological evaluation, and residue safety assessment. Implementation of the HACCP system became an essential step for ensuring the safety of milk and milk products. 

In 1997, based on the current scientific knowledge, the IDF introduced the so-called integrated system for the detection of antibiotics, sulfonamides, and inhibiting substances in raw and heat-treated milk. The integrated system proposed to control the presence of antimicrobial residues through a combination of multiple methods; at the same time, it defined the responsibilities of all participants of the dairy chain (primary milk producers, processors, and supervisory authorities) [21,22].

Due to the ever-expanding spectrum of veterinary medicines used in food animal therapy, the residues of which may be present in raw as well as processed foods of animal origin, the methods for the detection of antimicrobials have gradually changed. In order to prevent contaminated products from entering the food chain, a number of screening methods with sufficient sensitivity to a wide range of substances have been developed or modified. 

The review aims to provide a comprehensive overview of screening methods developed for the detection of antimicrobial residues in the context of their historical development up to the present day. It explains the specific principles and uses of these methods and highlights their pros and cons. The review provides useful information on screening methods (plate diffusion methods, commercially available microbiological inhibitory methods, rapid specific tests) that can be used on farms, in the dairy industry, and in milk quality control laboratories for the detection of antimicrobial residues. 

## 2. Methodology

The data were collected in several subsequent steps aiming to provide the reader with a complex overview of the topic. In the first stage, the Web of Science (Science Citation Index Expanded, Emerging Sources Citation Index, Conference Proceedings Citation Index), then other databases PubMed, CrossRef, Elsevier, Research Gate, Scopus, and Google Scholar were searched. The search strategy was designed to include initially a wide spectrum of relevant literature (original research studies, reviews, monographs, conference proceedings), using combinations of keywords with Boolean operators to ensure consistency and completeness of the review. The search was not time-limited to allow the explanation of the historical context and contemporary perspective.

In the next phase, specific keywords were used to collect information on individual groups of methods (e.g., keywords: “plate diffusion method”, “microbiological inhibition method”, etc.) or specific methods (e.g., Delvo test, BR test, etc.). 

To ensure the comprehensive and up-to-date character of the review, detailed information on specific methods was also retrieved directly from the manufacturers’ websites. 

Last but not least, publications of a legislative and technical nature served as another important source of data. This included in particular publications issued by the International Dairy Federation (IDF Bulletins), EU legal documents (EUR Lex), ISO standards, and Codex Alimentarius documents.

## 3. Requirements on Methods for Detecting Antimicrobial Residues

With the increase in the veterinary use of antimicrobials, it became necessary to develop a methodical framework for analysis, evaluation, validation, and comparison of data obtained using different tests as well as data from different studies. This was established by EN ISO 13969:2003 [(Milk and milk products—Guidelines for a standardized description of microbial inhibitor tests [23] and EN ISO 18330:2003 [(Milk and milk products-Guidelines for the description of immunoassays or receptor assays for the detection of antimicrobial residues [24]. The advancements in analytical chemistry led to a new approach, setting performance criteria and methods for validation of both screening and confirmatory methods. Within the European community, it was necessary to ensure the quality and comparability of analytical results obtained by different laboratories approved for official residue control in different countries. The objective should have been achieved in particular by using methods that were validated by general procedures on the basis of performance criteria and by ensuring consistency with generally accepted standards. For these reasons, Commission Decision 2002/657 was issued [25], laying down rules on the analytical methods to be used for the testing of official samples and specifying common criteria for the interpretation of analytical results. 

In 2010, the European Union Reference Laboratories (EU-RL) distributed a guidance document that supplemented the Commission Decision 2002/657/EC on the validation of screening methods. The objectives of this guideline document were to define (i): the minimum requirements to be fulfilled by the initial validation (in the ‘originator’ laboratory); (ii) criteria that are necessary to determine whether screening methods can be transferred to another laboratory and under what conditions; and (iii) the minimum requirements to be fulfilled by the abridged validation (in the ‘receptor’ laboratory) [26]. 

Specific EU legislation outlines the laboratory analysis and correct interpretation of results. Commission Implementing Regulation (EU) No 2021/808, repealing Commission Decision (EC) No 2002/657, specifies the performance characteristics that need to be determined for analytical methods. Screening and confirmatory methods used for the determination of antibiotic residues must meet the performance criteria and other requirements for analytical methods set out in the annex to the regulation and must be validated in accordance with the requirements laid down in that regulation. Qualitative screening methods must meet the criteria set in the Decision (EC) No 2002/657 for individual performance characteristics, namely detection capability (CCβ), selectivity/specificity, stability, and ruggedness. Screening methods are used as first-tier methods for the detection of the presence of antibiotic residues in raw foodstuffs of animal origin. The Regulation also defines the term “screening method” as “a method serving for screening of a substance of class of substances at a required level” [27].

In 2021, The International Organization for Standardization (ISO) published the Technical Specification ISO/TS 23758 | IDF RM 251 [28] ‘Guidelines for the validation of qualitative screening methods for the detection of residues of veterinary drugs in milk and milk products’. This international guideline described general workflows and protocols for the validation and verification of qualitative screening tests for the detection of residues of veterinary drugs in liquid milk (pasteurized, UHT, and reconstituted milk powders and whey protein extracts). This guideline was intended to be useful for manufacturers of screening test kits, laboratories validating screening methods or tests, competent authorities, and dairies or end users of reagents or tests for the detection of veterinary drug residues in milk products. 

## 4. Microbiological Inhibition Methods

The principle of microbiological inhibition methods builds on a common property of antimicrobials—on their ability to inhibit the growth of microorganisms. The methods are based on a specific reaction between the sensitive bacterial strain and the antimicrobial agent present in the sample, allowing qualitative or semi-quantitative determination of the presence of the antimicrobial. This principle, however, also implies that these methods are only able to detect antimicrobials or metabolites showing antimicrobial activity [2,29].

### 4.1. The First Microbiological Inhibition Methods

Between 1946 and 1961, multiple studies aimed to develop these methods using various bacterial strains sensitive to antimicrobial residues. The methods used the ability of selected bacterial strains to grow in agar medium (endpoint: turbidity), form metabolites (endpoint: e.g., acid production), and produce enzymes (the activity of which can then be measured based on dye reduction, such as methylene blue, resazurin, triphenyl tetrazolium chloride) [7]. The earliest microbiological inhibition methods were based on inhibiting the growth of lactic acid bacteria strains, which were later replaced with the spores of the genus *Bacillus*. Methods for the detection of antibiotic residues on other microbial strains have been developed, such as *Streptococcus lactis* [30,31], Streptococcus thermophilus [32,33,34,35,36,37], Bacillus cereus var. mycoides [38], or Bacillus stearothermophilus [39,40]. Some studies recommended the use of multi-strain starter cultures for the detection of residues in milk. Ruehe [41] proposed a simple test for the detection of penicillin in the milk. In that test, a 10 mL milk sample was heat-treated (79.4 °C, 5 min) and cooled to 22.2 °C. After that, it was inoculated with 1 mL of the starter culture. If the sample contained no penicillin, milk protein precipitation and formation of coagulum occurred within 10 h. Krienke [42] used a buttermilk starter to prove the presence of antibiotics in milk. Yogurt culture was considered the most sensitive starter culture, sensitive enough to detect very low concentrations of penicillin in milk [43,44]. 

### 4.2. Plate Diffusion Methods

#### 4.2.1. Development of the Plate Diffusion Methods

The introduction of plate diffusion methods, initially using the application of a sample using cylinders (cylinder plate method) constituted significant progress in the development of microbial inhibition assays. One of the first studies published on this topic [45] described the use of this method in human medicine. Studies on the use of the cylinder plate method and its modifications for the detection of antimicrobials in milk followed [46,47,48]. In 1944, Foster a Woodruff [46] described the cylinder plate method for the determination of penicillin residues in detail. The method used a suspension of spores of *Bacillus subtilis* for inoculation of an agar plate at a density of 15 × 10^3^ CFU/mL. Thirteen milliliters of thus inoculated agar were placed in each Patri dish. Subsequently, sterile glass cylinders (12 mm long, diameter of 8 mm) were heated, placed on the agar surface, and filled with the sample or standard penicillin solution. Following the incubation (30 °C for 12–16 h), the size of the inhibition zone of the sample was compared with that of the standard penicillin solution. The authors also proposed the possibility of a sample application using paper discs. The preparation of a standard suspension of the *B. subtilis* strain that could be stored in the refrigerator for an unlimited period of time was another methodical step crucial for the widespread development of plate diffusion methods. 

Initially, *S. aureus* and *B. subtilis* were the recommended testing strains for the cylinder plate method [45,46,47]. Later, additional strains were proposed, such as *Sarcina lutea* (penicillin detection), *Bacillus cereus* (streptomycin detection), *Bacillus cereus* var. *mycoides* (chlortetracycline), Group A *Streptococcus* (penicillin), or *Bacillus subtilis* (ATCC 6633) (streptomycin) [49]. In 1955, the cylinder plate method became an official FDA method [7]. 

The use of sterile paper discs for sample application brought a number of advantages and became another significant improvement in the application of plate diffusion methods. The first studies proposing the use of paper discs instead of cylinders came in about 1944 [50,51,52,53,54]. By 1961, most of the research in this area was completed. Drury [55] developed a disc-plate method that has served for the detection of antibiotic residues in milk for many years. That procedure employed Difco whey agar inoculated with a suspension of *B. subtilis spores*, at a final concentration of 250,000 CFU/mL agar. Ten milliliters of thus inoculated agar were applied per Petri dish. Paper discs (0.25 in) soaked with milk were placed on the surface of the seeded agar. Incubation at 37 °C for four hours followed.

The following years saw many studies utilizing the plate diffusion method with *B. subtilis* as a test strain for the determination of penicillin residues in milk. Several changes in the procedure were proposed to improve the method’s sensitivity and speed [56,57,58,59].

Wolin and Kosikowski [60] built upon results of studies showing that in the disc-plate method, even antimicrobial-free milk could have produced inhibition zones. These were apparently associated with the native antibacterial activity of fresh milk. Hence, raw milk resulted in two clearly defined inhibition zones (designated as primary and secondary) in a disc-plate method utilizing *B. subtilis* strain.

The discovery of the suitability of *B. stearothermophilus* as a test strain in microbial inhibition methods constituted another major improvement. Igarashi et al. [39] described a disc-plate method employing *Bacillus stearothermophilus* ATCC 7954 for ATB determination in various matrices. The use of *B. stearothermophilus* introduced multiple advantages: the thermophilic strain showed rapid growth (incubation 75 min) at 61–62 °C and high sensitivity to penicillin G (0.005 IU/mL milk). A year later, Igarashi et al. [40] described a colorimetric method with an even higher sensitivity (0.002 IU penicillin), based on an addition of a colorimetric redox indicator (2,3,5 triphenyltetrazolium chloride) to the agar medium. The same method was able also to detect oxytetracycline at concentrations of <1 µg/mL. The authors recommended heat treatment of the milk before testing, which reduced the incidence of false positive results. 

The introduction of a 4-plate diffusion test known as the Frontier Post Test in 1980 by Bogaerts and Wolf was another milestone in the field [61]. Despite being over forty years old, this method remains part of multi-plate diffusion methods for the determination of antimicrobial residues in foods and foodstuffs of animal origin. The method consisted of three plates with agar inoculated with a suspension of *B. subtilis* at three different pH values—6, 7.2, and 8. The sensitivity of the pH 7.2 agar to sulfonamides was increased by the addition of a trimethoprim solution. The fourth plate contained agar at pH 8 inoculated with a suspension of *Micrococcus luteus*. 

In 1993, the IDF published a Special Issue of its Bulletin [62] focusing on the importance and toxicological evaluation of inhibitory residues in milk and methods for residue detection. Plate diffusion methods were included among the microbiological inhibition screening methods and the following plate methods were recommended as preliminary confirmatory tests: a three-plate method with *Bacillus stearothermophilus* var. *calidolactis* C 953, *Bacillus subtilis*, and *Bacillus megaterium* and a six-plate method with *Bacillus cereus*, *Bacillus subtilis*, *Sarcina lutea*, *Escherichia coli*, and *Bacillus stearothermophilus* var. *calidolactis* [11].

Since the 1990s, many studies have been conducted to determine the sensitivity of plate methods to different antibiotics relative to established MRLs or to improve the sensitivities to selected antibiotics [63,64,65]. Some of them focused on the development and validation of new methods allowing the detection of specific groups of antimicrobials. One group of such novel antimicrobials of interest, quinolone chemotherapeutics, started to be used in veterinary medicine by the end of the 20th century. The plate method with *E. coli*, developed by Ellerbroek [66], later became a part of the multiplex diffusion method for the detection of antimicrobial residues in milk [65].

In 1996, Aureli et al. [67] introduced a post-screening multi-plate agar diffusion method allowing a closer identification of some antibiotic and sulfonamide groups in milk. This method used three different agar media and two test strains (*Bacillus stearothermophilus* var. *calidolactis* and *Bacillus subtilis* ATCC 6633) to test for penicillins, cephalosporins, sulfonamides, and streptomycin residues. A closer identification of antibiotics was achieved by adding solutions of para-aminobenzoic acid, L-cysteine, cephalosporinase, and penicillinase to the medium. 

In 1999, Nouws et al. [63] described a multi-plate system consisting of seven plates with six different test strains for the detection of antimicrobial residues in milk. Their study aimed to construct a multi-plate system that would allow the detection of a wide range of antimicrobials at levels close to the established MRLs (Table 1). 

The Community Reference Laboratory (CRL) in Fougères worked on enhancing the existing microbiological screening methods, aiming to improve the sensitivity to certain antibiotics as well as the accuracy of the determination for subsequent confirmatory methods. The STAR method (Screening Test for Antibiotic Residues) was developed for screening antibiotic residues in milk and muscles [68]. The STAR method consists of five test plates, each of which is preferentially sensitive to a selected group of antimicrobials (Table 2). 

#### 4.2.2. The Use of Plate Diffusion Methods at Present

To this day, plate diffusion methods constitute an important part of the system for the control of antimicrobial residues in the milk chain worldwide. Similar to most other microbiological inhibition methods, plate diffusion methods are important screening methods capable of detecting a wide range of antibacterial substances with different chemical structures. At the same time, they allow the group identification of antibacterials, finding use as post-screening confirmatory methods as well [65,69]. The STAR method is the recommended one in EU countries [65]. However, when comparing studies, it is obvious that the exact procedures differ among studies, which results in differences in the sensitivity to antimicrobials. Navrátilová et al. [70] described a multi-plate diffusion method consisting of six plates: three plates with *Bacillus subtilis* strain BGA CCM 4062 (agar; pH 6, 7.2, and 8); one plate with *Kocuria rhizophila* strain CCM 552, one plate with *Geobacillus stearothermophilus* v.c. C953 strain CCM 5965, and one plate with the *Escherichia coli* strain CCM 7372. This method is used in the Czech Republic as the reference method for the determination of residues of antimicrobial substances in raw foodstuffs and foods of animal origin [71]. Kirbiš et al. [72] aimed to develop a plate diffusion method for detecting residues of antibiotics from the groups of macrolides, aminoglycosides, cephalosporins, and tetracyclines. The method was based on the STAR test with the addition of two confirmation solutions. Confirmation solutions (magnesium sulfate and cephalosporinase) help to distinguish between groups of antibiotics which cause positive result on the same plate. 

In the USA, two plate diffusion methods are used. The cylinder plate method, using *Micrococcus luteus* as the test organism, is the official method for quantitative detection of β-lactam residues. The paper disk method using *Bacillus stearothermophilus* is the official method for qualitative detection of inhibitory substances in milk. It is a modification of the method approved by the International Dairy Federation for the qualitative detection of penicillin in milk [73].

Published studies show that plate diffusion methods are used in many regions. A study conducted in Iraq [74] focused on the occurrence of antibiotic residues in cow’s and buffalo’s milk. Antimicrobial residues were tested using plate diffusion methods with *B. subtilis* employing two types of sample application—well diffusion assay and disk diffusion assay. Plate diffusion methods utilizing *B. subtilis* are used as screening methods also in India, where raw milk samples from the pharms were screened using this method and positive samples were subsequently analyzed using the Charm ROSA test to differentiate between the residues of tetracycline, ß-lactams and enrofloxacin at non-permitted concentrations [75]. 

#### 4.2.3. Pros and Cons of Plate Diffusion Methods

As implied by published studies, the sensitivity of agar diffusion methods can be influenced by multiple factors, such as the composition and pH of the agar medium, its amount in the Petri dish (i.e., depth of the agar medium), the selected test strain, the concentration of cells or spores in the test medium, the method of sample application (paper discs, punch-holes, stainless steel cylinders), the amount or composition of the investigated sample, etc. [69]. The sensitivity of plate methods varies significantly with the type of the antibacterial agent. As the test strains do not have the same sensitivity to all antibacterial agents, a multi-plate system with multiple test strains is used. Still, the sensitivity of the detection of individual antibacterial agents differs [5] as confirmed by the results of studies discussed below. Nouws et al. [63] found that out of 48 tested antimicrobials, the multi-plate method failed to detect MRL concentrations of sulfanilamide, cefquinome, spectinomycin, and colistin. When validating the STAR method, Gaudin et al. [65] showed that out of 66 antibiotics tested, 21 antibiotics were detected by the STAR method at concentrations ≤ MRLs, while 27 more could only be detected at levels ranging from the established MRL to four times the MRL. In 14 antibiotics, the method sensitivity was even lower, ranging between 4 times and 150 times MRL. The method was shown to have sufficient detection capabilities for certain aminoglycosides (dihydrostreptomycin, streptomycin, kanamycin, spectinomycin) and other antibiotics (cephazolin, cefacetrile, flumequine, colistin, bacitracin, novobiocin, S-methazin, S-thiazole, S-methoxypyridazine). Navrátilová et al. [70] determined the sensitivity of their multi-plate method to residues of cephalosporins, for which MRLs are established in milk (ceftiofur, cefoperazone, cephalexine, cephazoline, cephalonium, cephapirine, cefquinome). Their results demonstrated the differences in sensitivity of the individual plate methods to selected cephalosporins. However, the multi-plate method was sensitive enough to detect most of the tested cephalosporins but cefquinome, could not be detected at levels close to MRL.

Another disadvantage of inhibition methods lies in their inability to detect the presence of metabolites that do not show bacteriostatic/bactericidal effects or non-hydrolyzed conjugates of antibacterial substances. Hence, plate diffusion methods may fail to provide complete information on the analyzed sample. In other words, sample negativity does not always prove the absence of veterinary drugs as not all drugs (and their metabolites) show antibacterial activity [69]. On the other hand, antibacterial substances naturally occurring in milk may also in some cases show inhibitory effects interfering with the assay, yielding false positive results [2]. The disadvantage of these methods lies in their long incubation period (18–24 h) along with their laboriousness and high demands on the procedure, preventing their use in primary milk production and the dairy industry. 

### 4.3. Commercially Available Microbiological Inhibition Methods

Despite some disadvantages, commercially available microbiological inhibition methods have been an important part of screening for antimicrobial residues in milk since the 1970s, mainly due to their ability to detect the presence of a wide range of antimicrobials. Antimicrobials reduce or completely interrupt the growth of the test strain, thus inhibiting the bacterial metabolic activity, which is subsequently measured using color indicators based on acid-base or redox processes. The color of the indicator (typically pH indicator–bromocresol purple) keeps changing in reaction to the acidic metabolic products of the growing test strain. Where redox reactions are used, the growing test culture reduces the indicator (brilliant black), which causes color change [5,29,76].

#### 4.3.1. Development of the First Commercially Available Microbiological Inhibition Methods

Kraack and Tolle developed the first broad-spectrum microbiological inhibition test, the Brilliant Black Reduction Test (BR-Test), in 1967 [77]. Their test was the first one to use the combination of diffusion agar plate test and color change. BR-Test was first produced in 1968 and as soon as 1969, it was introduced for microbial residue detection in the dairy industry. It was modified several times, with a temperature increase from 55 °C to 65 °C, and became simpler to perform and faster as the pre-diffusion stage was left out. The incubation time was less than three hours. Moreover, the range of detectable compounds grew and the test sensitivity improved [78].

In the 1970s, the first version of the Delvotest was developed to detect penicillin in milk. In 1975, van Os et al. [79] described a screening microbiological inhibition test using a *Bacillus stearothermophilus* var. *calidolactis* strain for the detection of penicillin in milk. Tablets containing nutrients and a pH indicator (bromocresol red) were added, together with the investigated milk sample, into vials containing agar medium inoculated with spores of the test strain. The vial was incubated for 2.5 h in a water bath at 63–66 °C. The test was based on agar diffusion. The nutrients, pH indicator, and antibiotic (if present in the milk) diffused into the agar medium, which was originally purple in color (presence of bromocresol purple). If the sample did not contain antimicrobial residues, the test strain grew, lowering the pH of the medium and subsequently changing the color of the medium from purple to yellow. The test showed high sensitivity to penicillin residues for both individual and bulk samples. At concentrations of 0.002 IU penicillin/mL or lower, the results were negative in all samples. Penicillin concentrations of 0.003, 0.004, and 0.005 IU/mL yielded mostly negative, dubious, and positive results, respectively. 

The first version of the Delvotest (Delvotest P, Gist-brocades BV, Delft, The Netherlands) came to the market in 1975 and has become one of the best-known and most widely used microbiological inhibition tests [2]. Development of other microbial inhibition tests followed. The production of a new method for the detection of antibiotic residues in milk, Valio T101 Method (T101 test), started in 1988. It utilized *Streptococcus thermophilus* that was used as a starter strain in some dairy products (Swiss cheese and yogurt) and, as it turned out, this specific “dairy strain” had the ability to successfully detect a wide range of antibiotics. Since 1989, based on good experience with this test in the dairy industry in Sweden and Finland, it has been distributed and used on farms as well [80]. 

In 1991–1993, Charm Sciences Inc. (Lawrence, KS, USA) introduced new methods for the determination of antimicrobial residues in milk. One of these tests, a broad-spectrum microbiological inhibition test, came in two formats: the Charm Farm Test (CFT) for testing individual milk samples (single assay format) and the AIM 96 (96-well microplate) test for testing larger sample sets [81,82]. 

#### 4.3.2. History of Some Commercially Available Microbiological Inhibition Tests and Their Current Variants

According to the IDF, qualitative methods have been defined as initial screening methods intended for the detection of positive (i.e., containing residues of antibiotics, including sulfonamides) samples. Most qualitative methods used *B. stearothermophilus var. calidolactis* ATCC 10149 as a test microorganism and were based on the principle of agar diffusion [11]. In its bulletin No. 258 (1991), IDF included the commercially available microbiological inhibition methods among screening tools for the detection of antibiotic residues in primary milk production [18]. Microbiological tests have been used for a long time as screening tests to detect residues in raw milk. There are numerous commercially available microbiological inhibition methods from various suppliers. The most used tests include the BR-Test (Analytik in Milch Produktions-und Vertriebs-GmbH, München, Germany), Delvotest^®^ (DSM Food Specialties, Delft, The Netherlands), Eclipse test (ZEU-Inmunotec SL, Zaragoza, Spain), Charm test (Charm Sciences Inc., Lawrence, KS, USA), and CMT-Copan Milk Test (Copan Italia s.p.a., Brescia, Italy). History of some tests and their current variants are described below.

- **BR-Test** (AiM-Analytik in Milch Produktions-und Vertriebs GmbH, München, Germany)

The BR-Test is a microbiological inhibition test frequently used for the detection of antimicrobial residues in milk, utilizing the *Bacillus stearothermophilus* var. *calidolactis* C953 test strain. The first version of BR-Test was highly sensitive to benzylpenicillin residues but poorly sensitive to sulfonamide and tetracycline residues. In the following years, new BR-Test variants were developed (BR-Test, BR-Test AS, BR-Test Blue Star, BR-Test AiM). Adriany et al. [83] introduced BR-Test modifications with improved sensitivity to sulfonamide and tetracycline residues. This improvement in sensitivity was achieved by adding antifolates (trimethoprim) which were proven to increase the antimicrobial activity of sulfonamides. Hence, 50 µg/mL of trimethoprim or a substance of equivalent effect was added to the agar medium (Antibiotic Medium no. 1). Moreover, the agar medium itself was found to contain high concentrations of sulfonamide antagonists and antifolates (p-aminobenzoic acid, folate, thymine, and thymidine), which reduced the sensitivity of the BR-Test to sulfonamides. Hence, other media not containing these compounds were tested; the best results were achieved using Mueller-Hinton agar enriched with 500 µg/mL EGTA (ethyleneglycol-bis-(b-amino-ethyl ether)-N,N,N,N-tetra-acetic acid) and pyrimethamin (800 ng/mL) and inoculated with a reduced spore count (5 × 10^6^–1 × 10^7^ CFU/mL). This modification allowed the detection of sulfonamides and benzylpenicillin at MRL concentrations (100 µg/kg and 2 µg/kg). However, the sensitivity of the modified assay, the BR-Test AS test, became insufficient for oxytetracycline (1500 µg/kg; the original BR-Test limit of detection was 1000 µg/kg). Tetracyclines form chelate complexes with bivalent ions, such as calcium ions in milk, and lose their antimicrobial activity. The addition of EGTA can improve the situation as the bond of EGTA to bivalent ions is stronger than that of tetracyclines. However, the amount of EGTA that has been originally added to agar to increase sensitivity to sulfonamides was insufficient to achieve this result. Therefore, another 20 µL of EGTA solution (50 mg/mL EGTA, pH 8.0) was added to the milk sample in each well of the BR-Test microplate, which significantly improved the sensitivity of the test to tetracyclines (detection limit of oxytetracycline 400 µg/kg), although it was still insufficient to detect MRL concentrations (MRL oxytetracycline = 100 µg/kg). 

A variant of the BR-Test AS (BR-Test “Blue Star”) has been officially adopted and used for raw milk testing in Canada. In this test, the concentration of spores in the detection medium was further reduced, which led to an improvement in sensitivity to sulfadimidine to <0.04 µg/mL milk. In addition to these modifications, two other variants, BR-Test 6 and BR-Test 7, were developed. Adjusting the pH from the original pH 8 to pH 7 increased the sensitivity of the test to aminoglycosides and sulfonamides. Similarly, the detection of tetracyclines was improved by lowering the agar pH from 8.0 to pH 6.0. It was considered necessary to use all three BR test-systems simultaneously (BR-Test 6. BR-Test 7, and modified BR-Test AS). This combined BR-test system allowed the detection of all antimicrobials at the required concentrations, except for chloramphenicol and kanamycin [78].

At present, the following variants of the BR-Test (BRT; Analytik in Milch Produktions- und Vertriebs-GmbH, München, Germany) are produced:

*BRT hi-sense*—a test kit for detecting inhibitors such as antibiotic residues in cow, sheep, and goat’s milk. The test shows a high sensitivity to beta-lactams, cephalosporins, tetracyclines, sulfonamides, tylosin, gentamycin, and neomycin at levels below the established MRLs (EU). The manufacturer states that BRT hi-sense is not sensitive to naturally occurring antimicrobials in milk, making it specific to detect the presence of antibiotic residues. The test can be used to detect residues even in high-fat and low-pH milk samples. The test is available in 2 formats: (i) as 48 individual tests in plastic tubes and as a box with five 96-well plates containing twelve strips of eight wells that can be broken down into strips of eight or even to individual wells that can be incubated separately. Assay time is 3 h 15 min ± 15 min.

*The BRT Inhibition Test* shows high sensitivity to beta-lactam antibiotics, which it detects at concentrations below or close to the MRLs (EU). The incubation time of the test is 2 h 15 min ± 30 min. The test is recommended for testing milk at the point of production, i.e., on the farm and in dairy plants. The test is available in 3 formats: single tubes or plates with separable rows (strips) or separable microtubes. The assay time is 2 h 15 min ± 30 min. 

*BRT MRL Screening Test* allows the detection of all beta-lactam antibiotics at concentrations below the established MRLs. The test is recommended for the screening of residues of beta-lactam antibiotics and cephalosporins in individual or pooled milk samples at farms. It can be used to check for residues in cow, goat, and sheep’s milk. Similar to the previous, the test comes in the form of individual tubes or plates with separable lines (strips) and/or microtubes. In addition to the improved sensitivity to beta-lactams, it also determines other antibiotics from the sulfonamide, macrolide, and aminoglycoside groups at MRL concentrations with an assay time of 2 h 15 min ± 30 min [84,85].

- **Delvotest^®^** (DSM Food Specialties, Delft, The Netherlands)

Delvotest^®^ P has been commercially produced in the form of vials for testing individual samples or small numbers of samples as well as in the form of microtiter plates for testing large numbers of samples. A color change from purple to yellow due to the formation of acid during incubation at 64 °C for 2.5 h indicates a negative result.

The Delvotest^®^ SP developed later was able to detect a wider range of substances, especially sulfonamides, and had improved sensitivity to tylosin, erythromycin, neomycin, gentamicin, trimethoprim, and other antimicrobials. Delvotest^®^ SP also allowed the detection of penicillin G at concentrations of 0.003–0.004 IU/mL. 

Evaluation and validation of the subsequently developed Delvotest^®^ SP-NT (sulfur-penicillin no tablet test) in accordance with ISO/IDF 183 requirements was presented by Stead et al. [86]. The Delvotest^®^ SP-NT differed from the previous test in incubation time, sensitivity, and the fact that nutrients were not added in the form of tablets but were a part of the agar medium. In both the tube and microplate forms, it was able to detect a wide spectrum of antibiotics at concentrations ≤ MRL (penicillin G, ampicillin, amoxicillin, cephapirin, ceftiofur, cloxacillin, sulfadiazine, and neomycin); however, the detection limits for oxytetracycline and erythromycin were 2–3 fold higher than their established MRLs. The microplates were suitable for reading by a novel Delvotest^®^scanner (Delvo^®^ Scan) technology approved by AOAC (Association of Official Agricultural Chemists). Delvotest^®^ SP-NT was recommended for screening for a wide range of milk samples—samples with different fat contents, somatic cell counts, as well as goat’s milk. It, however, could not be used for testing low-pH milk.

The Delvotest is currently available in the following variants, which can be used for testing individual milk samples (vials) as well as larger amounts of samples (microplates): 

*Delvotest^®^ P*—broad spectrum test.

*Delvotest^®^ T*—this method is a standard diffusion test for the qualitative detection of antibacterial substances such as penicillins, tetracyclines, sulfonamides, cephalosporins, macrolides, aminoglycosides, and lincosamides in raw milk. The test, however, does not detect the presence of quinolones in milk. The test supports testing of both individual and commingled raw cow’s milk samples. It comes in both tube and 96-well plate formats. Incubation of the test tubes can be done using either a water bath or a manufacturer-supplied incubator (Mini S block heater). Microplates can be assessed using either of the following three methods: visually, with the Delvo^®^Scan device, or automatically in the DAS (Delvotest^®^ Accelerator Smart) system [87].

*Delvotest^®^ SP-NT*—a broad-spectrum test detecting the main antibiotic groups, is available as test tubes or 96-well plates. The ampoules were designed for small-scale screening of individual milk samples for on-farm use, the 96-well version allows bulk testing in dairy plants, dairy industry, and quality control laboratories. The SP-NT DA system has been validated by Hennart and Faragher [88]. The Delvotest SP-NT DA Accelerator is an automated system in which the plates with analyzed samples are placed into an incubator equipped with DAS, which automatically detects the end of the incubation and reads the results. 

The sensitivities (LOD) of the Delvotest^®^ T and Delvotest^®^ SP-NT to the selected antibiotic species as reported by the manufacturer are shown in Table 3 [89].

- **Eclipse tests** (ZEU-Inmunotec SL, Zaragoza, Spain)

Eclipse tests by Zeu-Inmunotec, S.L., Spain, also count among the widely used tests. The range of these tests includes:

*Eclipse Farm*—a one-step kit producing results in about 2.5 h supplied in individual test tubes, interpretation by visual reading. 

*Eclipse 50*—a one-step kit producing results in about 2.5 h, supplied in the form of 96-well microtiter plates that can be split into individual wells. Interpretation by visual or photometrical reading. 

*Eclipse 100*—a two-step test taking about 3.5 h. Supplied in 96-well microtiter plates that can be split into individual wells. Interpretation by visual or photometrical reading.

*Screening Plus*—a kit test optimized for the detection of macrolides and aminoglycosides. It is performed in two steps that take about 3.5 h in total. Supplied in 96-well microtiter plates.

*Eclipse 4G&Comet32* is intended for detecting residues of 50 antibiotics from eight antibiotic groups in raw cow, sheep, and goat’s milk, supplied as microtiter plates. Highly suitable for testing large sample sets in supervisory laboratories and the dairy industry. The automatic system (Comet 32) enables incubation including its automatic termination and test results reading that can be observed on the display (qualitative positive/negative and numerical results). In a single run, up to 31 samples can be measured.

*Eclipse FARM-4G &Comet4* Eclipse FARM-4G &Comet4 is supplied in vials and intended for testing raw milk samples in primary production (quarter, individual, bulk milk samples) and in the dairy industry (tankers). The incubation time is 2.5 h. The Comet4 device is used to incubate the samples, automatically complete the incubation, and evaluate the test result, which is then automatically sent as a report via Bluetooth and/or Wi-Fi to the user’s smartphone. The user, therefore, does not need to be physically present during the incubation and result evaluation.

*Eclipse FARM &COMET8* also comes in vials and is intended for testing both raw and heat-treated milk. The test can be used to evaluate cow’s, buffalo’s, goat’s, and sheep’s milk and is intended for dairy farmers and milk processors. The COMET8 equipment can be used to perform the test, providing the same functions as the aforementioned Comet4 device [90]. The Eclipse test sensitivity compared to MRL is shown in Table 4.

- **Charm tests** (Charm Sciences Inc., Lawrence, KS, USA)

Charm Sciences Inc. (USA) has introduced the following broad-spectrum microbiological inhibition tests:

*Charm CowSide II Test* shows high sensitivity to beta-lactam antibiotics, sulfonamides, aminoglycosides, and especially tetracyclines. The test is supplied as individual ampoules along with an incubator. 

*Charm Blue Yellow II Test* is designed for the detection of antimicrobial residues in pooled raw milk, pasteurized, and ultra-pasteurized milk samples. The manufacturer states that the presence of higher concentrations of natural antimicrobials in abnormal milk, disinfectants, and substances used for sanitation of the equipment may cause false-positive test results [91]. The sensitivities of Charm tests to individual antimicrobials are shown in Table 4.

- **CMT-Copan Milk Test** (Copan Italia S.p.A, Brescia, Italy)

This is another broad-spectrum microbiological inhibition test produced in two formats—individual tests (vials) for analysis of a small number of samples (*CMT Single Test*) and in the form of multi-well microplates for larger-scale analyses (*CMT Microplate*). The agar is pre-seeded with spores of *Bacillus stearothermophilus* var. *calidolactis* and includes a fermentable sugar (glucose) and a pH indicator (Bromocresol Purple). It can be used for the detection of antimicrobial residuals in raw cow’s, goat’s, and sheep’s milk as well as in heat-treated and powdered milk [92]. Sensitivities of the CMT test to individual antimicrobials are shown in Table 4.

- **Other commercially available microbiological inhibition tests**

The range of broad-spectrum inhibition tests is wide. For example, we can name the Kalidos test (EuroClone S.p.A., Pero, Italy) suitable for the analysis of residues in raw cow’s, goat’s, and sheep’s milk. Similarly to most tests, it is available in the form of individual vials (Kalidos TB 100 tests) as well as of microplates (Kalidos MP-3 × 96 tests) [93]. Another test, Accuplus MAT5 (Ring Biotechnology Co., Ltd., Beijing, China) detects the presence of the residues of more than 34 antibiotics in milk [94].

#### 4.3.3. The Use of Commercially Available Microbiological Inhibition Tests at Present

Literature confirms that commercially produced microbiological inhibition tests are still among the most common methods used for the determination of antimicrobial residues in milk. Treiber and Beranek-Knauer [95] summarized the results of 73 studies on the determination of antimicrobial residues in products of animal origin published between 1999 and 2021. In these studies, the following methods were used for the detection of residues of antibiotics and their concentration in milk: ELISA (4×), U/HPLC (6×), GC (1×), LC-MS (4×), Inhibitor test (1×) and various test kits (12×). This confirms the success of commercially produced tests enabling a simple detection of a wide spectrum of antibiotics. Namely, the studies employed the following tests: the Charm Blue Yellow antibiotic residue test kit (Charm Sciences Incorporated, MA, USA), Copan test kit (Christian Hansen Company, Hoersholm, Denmark), Delvo SP^®^ test kit (SP Mini Kit, Delft, The Netherlands). Virto et al. [96] also suggests that commercially produced microbiological inhibition tests are used to detect residues in milk. The review summarized and evaluated the results of published scientific literature worldwide reporting on the presence of antibiotic residues in milk and dairy products. The methods applied in milk analysis included Charm II Blue Yelow, Charm AIM, Delvotest^®^, Delvotest SP^®^, Delvotest SP-NT^®^, Charm Blue Yelow, Copan test kit, and Eclipse 100. 

#### 4.3.4. Pros and Cons of Commercially Available Microbiological Inhibition Methods

Many commercially available microbiological inhibition methods have long held a place among the screening methods used to detect the presence of antimicrobial residues in milk. The reasons for the success of these methods include the simplicity of the procedure, reliability, sensitivity to a wide range of substances, relatively long shelf life, and low costs [2,6]. The tests, however, also come with limitations—they are qualitative, do not support the identification of specific antibiotics, their detection limits for many antimicrobials are unsatisfactory—for some antibiotics even several times higher than MRLs. The incubation time is much longer than in the case of rapid specific tests [2,69]. Moreover, the results of these methods can be influenced by various interfering factors (natural antimicrobials, somatic cell count, sample microflora, metabolic activity of microorganisms in milk, etc.) [6]. The presence of natural antimicrobials in milk (lactoferrin, lysozyme) can, under certain conditions, cause false-positive results. According to Beukers [97], both the contaminant residues and natural antimicrobials can diffuse into the agar and affect the growth of the test microorganism. However, lysozyme and lactoferrin can diffuse only into a thin superficial layer and, therefore, will not affect the growth of the test microorganism throughout the entire medium depth. This is caused by the fact that lactoferrin and lysozyme are strongly alkaline proteins and have a positive charge at neutral pH, which prevents their diffusion due to the interaction with sulfhydryl groups of agar, which have a negative charge. In the bottom part of the well/vial with the agar medium, therefore, the growth of the test strain is not affected by the natural inhibitors. This may lead to the seemingly curious result of a thin purple layer above a yellow agar layer even if the tested sample is negative for contaminating antimicrobials. 

Other studies have shown that individually, lactoferrin and lysozyme do not cause false positive results in milk unless present in non-physiologically high concentrations (mastitic milk, colostrum). However, their combination has been found to exhibit a synergistic effect. Still, where this synergic effect of both antibacterials is concerned, the ion-binding effects of agar cause false-positive Delvotest results only at lactoferrin and lysozyme concentrations of 0.7 µg/mL a 1000 µg/mL, respectively, which is much higher than commonly found in the milk of healthy milked cows [6,97].

The occurrence of false-positive results by these methods has also been related to increased somatic cell counts in milk [98]. The probability of false-positive results was also higher in sheep’s milk with lower dry matter content, high somatic cell count, and higher thiocyanate content [99]. On the other hand, low sample pH values (pH < 6.1) can lead to false negative results [6].

### 4.4. Rapid Specific Tests for the Detection of Antibiotics in Milk

Due to the increasing demands of milk producers and processors, various rapid specific tests have been developed and introduced into practice. These rapid tests provide qualitative or semi-quantitative results and many of them are portable, i.e., suitable for in situ use. Ideally, the results of these tests should be available within 30 min and sample preparation prior to analysis should be minimal. Currently, rapid tests are widely used in primary milk production where they can quickly provide the farmer with information on whether milk from an antimicrobial-treated cow is (after the withdrawal period) suitable for human consumption. In the dairy industry, these tests provide the milk processor with information on the health and technological safety of the product. In the laboratory, rapid specific tests can be used as post-screening tests for preliminary identification of the contaminants. Rapid specific tests detect molecular interactions between antibiotics and ligands (antibody or receptor protein) [100]. 

#### 4.4.1. Microbial Receptor-Based Assay

The first rapid test, Charm I test for the detection of β-lactam antibiotic residues, was developed in 1979. It became the first rapid assay with a duration of 15 min recognized by AOAC International (Association of Official Analytical Collaboration International) [2]. A few years later, in 1984, the Charm II test was developed, allowing the detection of a wide range of drugs—β-lactams, tetracyclines, aminoglycosides, sulfonamides, macrolides, chloramphenicol, and novobiocin.

Charm I and Charm II are microbiological competitive receptor methods for rapid detection and identification of antibiotics in milk. Receptor methods use a natural binding site in a microbial cell to form a bond with a specific substance (antibiotic), and a single bacterial cell can contain binding sites for multiple antibiotics. The binding reactions have a low dissociation constant, which is favorable for the specificity and sensitivity of the reactions. During the development of the Charm I assay, the ability of different species of microorganisms to bind beta-lactam antibiotics was tested and *B. stearothermophilus* was selected as the most suitable. Other strains of microorganisms provided more suitable receptors for other antibiotics. The β-lactam antibiotics were found to bind to receptors on/or near the surface of the microbial cell wall, whereas other antibiotics bound to receptors inside the cell, e.g., ribosomes. The Charm II assay uses the effect of competitive binding of the analyte vs. a ^14^C or ^3^H radiolabeled substance to the specific binding receptor or antibody (for the detection of tetracycline and chloramphenicol). These two radioisotopes are the most suitable for routine analysis, having a longer half-life and a low level of beta radiation. The amount of ^14^C- or ^3^H-labeled substance bound to the surface is measured based on their radioactivity using the analyzer in CPM (count per minute) units. The more analyte is present in the sample, the less ^14^C- or ^3^H-labeled substance is bound to the receptor or antibody and, therefore, the measured radioactivity drops with an increasing amount of contaminants. Charm methods give a result in 7–18 min [3,101]. Examples of Charm II tests for the detection of various types of antimicrobials are shown in Table 5.

#### 4.4.2. Receptor Binding and Enzymatic Assays

Methods using receptor proteins have also found application as early screening assays, particularly those intended for the determination of beta-lactam antibiotics. Beta-lactam-specific receptor proteins (penicillin-binding proteins, PBPs) forming a part of the bacterial cell wall have been successfully used in commercially available assays (e.g., Penzym test, Beta-Star test, SNAP test, Charm Safe Level test, and DELVO-X-Press test). PBPs have specific functions in the bacterial cell and possess transpeptidase, transglycosylase, and carboxypeptidase activity. Penicillin is a structural analog to the natural substrates of PBPs (D-alanyl-D-alanine-terminated peptides), namely the dipeptide D-Ala-D-Ala; therefore, PBPs react with the beta-lactam structure [103,104,105].

#### 4.4.3. Lateral Flow Immunoassay

The ever-increasing requirements for rapid and reliable detection of antibiotics in milk led to the development of new detection methods, such as Lateral Flow (Immuno)Chromatography—LFIA, which has been growing in importance in this field over the last two decades. Lateral flow immunoassays were first developed by the end of the 1960s. The first such test was designed for the detection of human chorionic gonadotropin (hCG) in urine. Subsequently, the application of LFIA has expanded from human medicine to other disciplines because it allows the detection of various molecules, such as tumor markers, pathogens, mycotoxins, heavy metals, pesticides, and pharmaceuticals. The analyte detection is based on a chromatographic system separating the components of a mixture based on the differences in their transport through the reaction membrane and an immunochemical reaction (antibody-antigen). The liquid sample containing the analyte of interest moves through a strip made of polymeric materials containing specific zones [106,107].

##### Use of LFIA for the Detection of Antimicrobials in Milk

Immunochromatographic tests can come in different formats—most commonly as strips for immersion into a liquid or plastic cartridges forming a cover for the strip. Currently, the strip format is the most widely used. The immunochromatographic strip consists of four parts: the sample application pad, the conjugation pad, the membrane containing the test and control lines, and the absorption pad [106,108]. The first part of the strip contains the sample application zone (sample pad), which is usually made of cellulose or fiberglass to slowly and continuously transfer the sample in a homogeneous manner to the conjugate zone. In some cases, the sample application zone is designed to include sample pretreatment—it may be saturated with pH-adjusting agents, salts, proteins, and/or surfactants that affect the rate of analyte diffusion through the membrane. It may also act as a filter to remove coarse particles from the sample. 

The next zone, the conjugate zone, is most commonly made of fiberglass, polyester, or cellulose and contains the adsorbed immobilized conjugate—a specific bioreactive molecule (antibody, antigen, receptor protein) labeled with an indicator. In this context, the word conjugation means the binding between the indicator molecule and the biorecognition molecule. The adsorbed conjugate should be chemically stable and should only be released from the site of fixation when reacting with the liquid sample. The visual detection of the analysis result is made possible by the use of dye markers—indicators in the form of nanoparticles (tens of nm). Gold nanoparticles are currently the most commonly used markers in immunochromatographic tests. They have an intense red color easily detectable by the naked eye. Besides gold nanoparticles, colored latex nanoparticles, carbon nanoparticles, etc. are also used. 

The membrane is one of the most important parts of the strip. It is often made of nitrocellulose, but other polymeric materials such as polyethylene or nylon are also used. The membrane material determines the sensitivity of the immunochromatographic test. The membrane is typically placed on a plastic reinforcement support because the materials from which it is made are very fragile and thin. The membrane material is selected in view of its ability to bind reagents well and the pore size. The membrane also contains a test line (detection zone) and a control line. At the end of the strip test, there is an adsorption pad ensuring that the liquid rises through the membrane, prevents backflow and, at the same time, captures any excess liquid [106,107,108,109,110]. 

For the detection of substances with low molecular weight (including antimicrobials), the competitive immunochromatographic design is used. They contain only one antigenic determinant on their surface, the so-called epitope, which is able to bind to the antibody binding site; for this reason, it is not possible to use a sandwich design [106]. 

The assay can have two layouts. If the first layout is used, the sample is applied onto the sample application pad. At conjugate pad, captured labeled analyte conjugate gets hydrated and starts flowing with moving sample. If the sample does not contain the analyte, the sample travels up the strip to the detection zone of the membrane (test line) containing the bound primary antibodies to analyte. The labeled analyte binds to the primary antibodies on the test line and to the secondary antibodies at the control line site. Both the test and control lines are colored red. If the sample is positive, the sample analyte and labeled analyte compete for binding sites on the antibody. 

In the second layout, the first stage of the procedure involves mixing the sample with the labeled primary antibody (dispensed at conjugate pad). The test line carries the protein-analyte conjugate, and the control line contains secondary antibodies. In this arrangement, the analyte (antigen) in the sample and analyte-conjugate which is immobilized at test line compete for binding to the labeled antibody. In both assay configurations, the results obtained correlate negatively with the analyte concentration. The more analyte in the sample, the weaker the staining of the test line [100,106]. 

In some LFIA methods, specific receptor proteins are used as biorecognitive molecules. As an example of such an assay, we can name the BetaStar^®^ Plus Test (Neogen Corporation, Lansing, MI, USA) for detecting beta-lactam antibiotics and ceftiofur in milk. The assay utilizes two detection systems: ceftiofur is detected through an anti-desfuroylceftiofur monoclonal antibody, whereas other β-lactams are detected through a β-lactam bacterial protein receptor. First, a mixture of the two conjugates (receptor protein-gold particle conjugate and an anti-ceftiofur antibody-gold particle conjugate) is incubated with a milk sample. If beta-lactam antibiotics are present in the sample, they bind to the receptor-gold particle conjugate, while ceftiofur and its metabolites bind to the antibody-gold conjugate. Subsequently, an immunochromatographic strip with two test lines in the detection zone (which will detect the receptor and antibody) is inserted into the sample, one with the bound beta-lactam antibiotic and the other with a bound ceftiofur metabolite. If the receptor has not been in contact with ß-lactams, the band will capture all the receptor molecules. This will result in a visible red band being formed in the test area (at the line with bound beta-lactam antibiotics). If no band is formed, the receptors have been blocked by ß-lactams. As the sample flows up the membrane, a red line forms at one or both of the test lines, with intensity inversely proportional to the amount of beta-lactam antibiotic and/or ceftiofur present in the milk sample.

Additional labeled reagents present in the conjugate preparation react at the control line to form another red line. The relative intensity of the test and control lines is determined using the AccuScan III Reader. A ratio of >1.0 for both test lines indicates a negative test result, while a ratio of ≤1.0 for the individual test lines indicates a positive test result for the respective antibiotic [111].

The LFIA tests can be evaluated visually by comparing the intensity of the color of the control and test lines. The advantage lies in a quick qualitative interpretation of the results as “positive” or “negative”. Moreover, manufacturers increasingly support results evaluation using reading equipment, which allows more accurate (semi-quantitative) analysis and archiving of results. In semi-quantitative determination, the strip reader records the intensities of the test and control lines and converts the ratio of staining intensity to numerical values [109]. Some suppliers provide software that allows processing of imagery taken using not just professional readers, but also ordinary cameras or even mobile phone cameras.

At present, a broad range of rapid specific tests based on the LFIA principle is available. Such tests can be used to detect specific antibiotics (e.g., TyloSensor for detection of tylosin in milk), to detect and simultaneously identify multiple antibiotics within one group (e.g., Charm Neomycin, Streptomycin and Gentamicin Test for Milk, 4 Aminosensor Milk), to detect the presence of antibiotics belonging to a single group or multiple groups. Examples of rapid specific tests for the detection of antimicrobial residues in milk are given in Table 6; Table 7 then shows the sensitivity of selected tests to antimicrobials.

##### Pros and Cons of LFIA

There are a number of advantages to using LFIAs. The most significant advantages for users include low cost, ease of device preparation, relatively long shelf life, simple and user-friendly operation, small sample volume, short time of analysis, sensitivity and specificity comparable to or better than those of other established methods, high potential of commercialization, easy integration with electronics, stability over a wide range of environmental conditions, negligible energy consumption, usually allowing sample application without pretreatment. Like all methods, however, LFIAs also have some disadvantages—they are mostly qualitative or semi-quantitative, their reproducibility varies from lot to lot, pretreatment of the sample may be required with some tests, which increases the time demand [109]. 

Many manufacturers focus on making the tests easy and user-friendly, facilitating their use in the field as well as in laboratories. Breeders need to use the tests on-farm; for many tests, portable mini-incubators are supplied, allowing on-site incubation and reading of the test. Nowadays, even incubators with automatic termination of incubation followed by the automatic evaluation of the result and data transfer using a mobile app are available, making the presence of the user unnecessary once the test is initiated.

Similarly, a portable reader may be used by milk processors, allowing milk testing during transport in a tanker and conveying the message to the dairy plant prior to arrival. Mobile apps paired with such readers support also data storage, management, and archiving, thus supporting the transparency of the process. In this way, such readers can be useful also in a laboratory.

## 5. Discussion

The presence of antimicrobial residues in milk can pose a serious threat to human health. Antimicrobial residues can have various toxic effects, including immunopathological, teratogenic, carcinogenic, and mutagenic effects, and can cause allergic reactions. In addition to toxic effects, antimicrobial residues have an adverse effect on the human microbiome, affecting its composition and thus its function. Another major global public health and food safety problem lies in the emergence and transmission of antibiotic resistance associated with antimicrobial residues in the human diet [116,117,118,119].

MRL is maximum level or concentration of a drug or chemical thought to be non-hazardous and permitted by the regulatory bodies in or on food or feed intended to be used for animal or human consumption at a specified point of time, known as MRL. Although efforts have been made to harmonize maximum residue limits worldwide under the aegis of World Trade Organization (WTO) and the Codex Alimentarius, MRLs still vary from one geographical location to another, the effort was not successful. Maximum residue limits of veterinary drugs may be defined at the national/regional or international level depending on the local food safety regulatory agencies and drug usage patterns. For example, FDA established so-called “safe” levels, EU established MRL. Table 8 shows current the MRL (EU) and Codex MRL values for selected antibiotics in milk [120,121].

As obvious from the recommendations and guidelines issued for stakeholders in the milk production chain listed below [122,123], screening methods have an indispensable role in the context of dairy chain safety regarding the presence of antimicrobial residues and, consequently, the promotion and restoration of public health of the population. Their role is summarized in the Codex Alimentarius definition: „Screening methods are qualitative or semi-quantitative in nature and are used as methods to identify the presence (or absence) of samples from a herd or lot which may contain residues which exceed an MRLVD or other regulatory action limit established by a competent authority”. They may be applied to a sample at the point of entry into the food chain, site of inspection, or on receipt of a sample at the laboratory to determine if the sample contains residues that may exceed a regulatory limit [123].

According to the IDF [122], prevention of milk contamination at the point of production starts with the correct and responsible use of antibiotics, including compliance with their withdrawal periods. In the case of the use of on-label drugs and compliance with the withdrawal period, milk from a treated dairy cow should be safe for the consumer. After the withdrawal period, drug residues in milk should be at concentrations not exceeding the established limits for the substance and its metabolites (MRLs). Nevertheless, some farmers carry out testing of individual samples for precautionary or commercial reasons. In cases where the antibiotics used ‘on-label’ have been correctly administered and a withdrawal period has followed, but the test is positive, the milk from the animal must continue to be withheld from the food chain. In the case of off-label administration, it is always recommended that, after the withdrawal period, farmers collect and test the milk of treated animals to ensure that their products (milk) do not contain drug residues at unauthorized concentrations and thus comply with the precautionary approach. This examination of individual milk samples before inclusion in the milk supply will also prevent economic losses or devaluation of the whole milk supply. Monitoring of residues of veterinary medicines is also an important critical control point (CCP) in the HACCP system in raw milk production. The choice of the most appropriate screening test depends on the specific circumstances, in particular on the antibiotics that have been applied. The detection limit of the chosen test/method for a given antimicrobial must be at or close to the established MRL. In primary production, both microbiological inhibition tests and rapid specific tests are appropriate, although both have their strengths and weaknesses. For example, it should be taken into account that microbiological inhibition tests may, under certain circumstances (especially when testing individual samples), give false positive results due to interfering factors (high concentrations of natural inhibitory substances, high somatic cell counts).

Bulk milk samples from farms are regularly tested in accredited milk quality control laboratories. The milk must meet the quality criteria laid down by the legislation and at the same time meet the quality requirements for reimbursement based on contractual requirements with the purchaser. Antibiotic residues are analyzed as one of the parameters for the toxicological safety of milk under current legislation [124] and are also an important quality parameter for setting the price of milk. Milk processors are responsible for implementing an efficient, robust, and affordable testing strategy. Most milk quality control laboratories use commercially produced microbiological inhibition methods/tests capable of detecting a wide range of substances, although the LODs for some substances are higher than the MRLs, some other substances are even not detectable using these methods [122].

According to the Commission Regulation 853/2004 [124], as amended, food business operators must establish procedures ensuring that raw milk is not placed on the market if it contains antibiotic residues exceeding the levels permitted by the Regulation itself, i.e., MRLs, for any of the substances listed in the Annex to the Commission Regulation (EU) No. 37/2010 [120]. The responsibility for the quality of the milk lies with the milk producer and, once received at the processing plant, with the processor. As required by legislation, milk processors must test all raw milk (raw material) received at a dairy plant for the presence of antimicrobial residues. In addition to food safety requirements, the presence of antimicrobial inhibitors in milk is a technological risk issue for milk processors in dairy fermentation processes. Milk must be safe and suitable for dairy processing (technological safety). The investigation is carried out by taking and testing tanker samples and the entire tank cannot be accepted for further processing unless a laboratory test confirms its safety. In the event of an unsatisfactory result, the milk must be excluded from further processing and properly disposed of. The dairy industry mainly uses rapid specific tests, allowing very quick checks for antimicrobial residues before the milk is accepted for further processing into dairy products. Such rapid analysis of all milk on receipt ensures that the processing plant meets the current EU legislation [122]. As mentioned above, a significant benefit of these rapid specific methods lies in the speed of results acquisition; on the other hand, these methods are so specific that they selectively detect only particular groups of antibiotics or specific substances. Thus, some antibiotics present in the sample may remain undetected.

Samples which test “positive” with the screening (qualitative) method are considered as suspect and are usually designated for further laboratory testing using confirmatory methods. Confirmatory methods are used to obtain the identity, chemical structure and concentration of antibiotics. In general, physico-chemical methods for the confirmation of antimicrobials are based on the chromatographic separation of residues, especially Liquid Chromatography (LC), followed by spectroscopic quantification, such as UV, fluorescence or mass spectrometry (MS). Despite the associated costs, mass spectrometric methods have been used in last years for very selective and specific multi-compound detection [121,122].

## 6. Conclusions and Future Perspectives

Screening methods for the determination of antimicrobial residues in milk have made significant advances since the 1980s, which has contributed to a major improvement in antimicrobial residue control programs in milk. With the ever-broadening spectrum of antimicrobials, the demand for screening methods capable of rapid, sensitive, and reliable detection of their residues keeps also increasing. The trend is also towards performing the tests on-site (on farms or in milk processing plants) by operators without specialized training instead of laboratory workers, which leads to most commercially available tests being offered in robust and user-friendly formats. The development of these methods can be seen, for example, in:

(1) the wide range of commercially available test formats, from test kits usable in situ to sophisticated laboratory instrumentation systems allowing large-scale testing in laboratories,
(2) screening methods that can be used to analyze for a wide range of antimicrobials, with some allowing the detection of a specific antibiotic, while others detecting entire groups of antibiotics simultaneously,
(3) manufacturers offering test kits compatible with automatic termination of incubation and test evaluation, with subsequent data transfer to a mobile app, or
(4) the availability of assays with different sensitivities reflecting specific requirements for maximum concentration levels of antimicrobials according to specific sets of limits (EU MRL, Codex MRL, FDA limits).

## Figures and Tables

**Table 1:** A multi-plate system developed by Nouws et al. [63].

Test Organism	Agar Medium/pH	Incubation Temperature [°C]/Time [h]	Sensitivity
*Bacillus stearothermophilus* var. *calidolactis* C 953	Plate count agar (Difco)/8.0	55/6	Β-lactam antibiotics, tylosin, rifamycin
*Bacillus cereus* var. *mycoides* ATCC 11778	Standard II Nähragar (Merck) + supplements/6.0	30/*	tetracyclines
*Bacillus subtilis* BGA (Merck art. 10649)	Plate count agar (Difco)/8.0	30/*	aminoglycosides
*Bacillus subtilis* BGA (sulfa plate)	Mueller Hinton agar (Oxoid)+ supplements/7.0	37/*	sulfonamides, dapsone, trimethoprim, bacquiloprim
*Micrococcus luteus* ATCC 9341	Mueller Hinton agar (Merck)+ supplements/8.0	30/*	macrolides, rifamycin
*Escherichia coli* ATCC 11303	Plate count agar/6.0	30/*	quinolones, colistin
*Staphylococcus epidermidis* ATCC 12228	Plate count agar/6.0	30/*	novobiocin, rifamycin

* Overnight incubation.

**Table 2:** The STAR method [68].

Test Organism	Agar Medium/pH	Incubation Temperature [°C]/Time [h]	Sensitivity
*Bacillus stearothermophilus* var. *calidolactis* ATCC 10149	DST medium/7.4	55 °C/12–15 h	sulfonamides and ß–lactam antibiotics
*Bacillus subtilis* BGA DSMZ 618	Antibiotic medium II. Difco/7.2	30 °C/18 h	aminoglycosides
*Bacillus cereus* ATCC 11778	Test agar Merck/6.0	30 °C/18 h	tetracyclines
*Kocuria varians* ATCC 9341	Test agar Merck/8.0	37 °C/24 h	makrolides and ß–laktam antibiotics
*Escherichia coli* ATCC 11303	Test agar Merck/8.0	37 °C/18 h	chinolones

**Table 3:** Limits of detection [µg/kg] of Delvotest^®^ T and Delvotest^®^ SP-NT available microbiological inhibitory tests [89].

Antibiotic	MRL-EU [µg/kg]	Delvotest^®^ T		Delvotest^®^ SP-NT	
		Plates	Ampoules	Plates	Ampoules
Amoxicillin	4	4	4	3	2
Ampicillin	4	3	4	2	2
Penicillin G	4	1	1	2	2
Cloxacillin	30	5	6	12	12
Cephalexin	100	20	20	45	45
Cefalonium	20	10	8	10	10
Cephapirin	60	5	6	2	2
Ceftiofur	100	20	20	20	20
Cefoperazone	50	40	40	30	30
Cefquinome	20	40	40	75	65
Oxytetracycline	100	80	100	250	300
Tetracycline	100	75	70	270	300
Sulfadiazine	100	50	40	50	65
Tylosin	50	35	35	40	35
Tilmicosin	50	60	60	30	30
Kanamycin	150	1.310	1.010	1.700	1.700
Neomycin	1.500	110	60	190	115
DH Streptomycin	200	800	500	700	700
Framycetin Sulp	1.500	110	>150	120	120
Trimethoprim	50	130	110	160	160

**Table 4:** Limits of detection of some commercial microbiological inhibitory tests [µg/kg] [90,91,92].

Antimicrobial Agents	MRL-EU [µg/kg]	Charm Blue Yellow II Test	Charm CowSide II Test	Eclipse 50	Eclipse FARM & COMET8	Eclipse FARM-4G & COMET4	Copan Milk Test
β-lactams							
Amoxicillin	4	2–3	3–4	4	4	3	2–4
Ampicillin	4	2–3	3–4	4	4	3	<2
Cephalexin	100	60–100	75–100	60	50	50	>45
Cefapirin	60	4–6	8–10	8	10	5	2.5–5
Cefalonium	20	10–15	15–20	20	20	5	-
Ceftiofur	100	50–100 *	50–100	100	50	25	50–100
Cefazolin	50	6–10	6–10	35	50	-	5–10
Cefoperazone	50	20–30	20–30	50	50	25	25–50
Cefquinome	20	40–60	40–60	150	-	20–40	30–100
Cloxacillin	30	10–20	10–25	30	30	25	10–15
Oxacillin	30	8–10	5–10	20	15	15	5–10
Penicillin G	4	1–2	2–3	3–4	3	2	1–2
Tetracyclines							
Doxycycline	0	25–75	25–75	100	-	100	150
Chlortetracycline	100	150–200	200–300	-	150	100	250–500
Oxytetracycline	100	75–100	75–100	100–150	100	100	250–500
Tetracycline	100	75–100	50–100	100–150	100	100–125	250–500
Sulfonamides							
Sulfadiazine	100	80–100	40–60	100	50	50	50–100
Sulfamethazine	100	75–125	75–125	150	100	100–125	100–200
Sulfamethoxazole	100	-	-	100	-	-	<50
Sulfapyridine	100	75–125	-	-	-	-	-
Sulfathiazole	100	25–75	-	100	50	75	50–100
Macrolides							
Erythromycin	40	100–150	75–100	200	120	80–100	>200
Tylosin	50	20–30	20–30	50	25	40	50–100
Tilmicosin	50	25–35	25–35	100	50	-	75–100
Spiramycin	200	400–500	300–400	>400	-	-	>2000
Aminoglycosides							
Dihydrostreptomycin	200	-	-	800	500	800	<1000
Gentamycin	100	75–100	75–150	400	50	150	100–500
Neomycin	1500	75–150	100–150	1500	200	500	500–2000
Kanamycin	150	-	-	>2000	-	-	-
Spectinomycin	200	-	-	2500	-	-	>300
Streptomycin	200	-	-	>2000	500	1.200	<1000
Lincosamides							
Lincomycin	150	100–150	75–150	300	150	150–200	150
others							
Trimethoprim	50	200–300	200–300	-	-	-	100–150
Dapsone	5	1–2	1–2	-	5	5	2–4

* Concentration listed are total parent and metabolites. The concentration for positive for parent ceftiofur only is 10–20 µg/kg.

**Table 5:** Charm II tests (microbiological receptor tests) for the detection of various antibiotic groups [102].

Test	Group of Antibiotics	Type of Reaction	Time per Analysis
Charm II (Streptomycin Type) Aminoglycoside Test	streptomycin-type aminoglycoside drugs	rapid microbial receptor assay	18 min
Charm II Beta-lactam (Sequential) Test	beta-lactams	rapid microbial receptor assay	12 min
Charm II Novobiocin Test	novobiocin	rapid microbial receptor assay	7 min
Charm II Sulfa Drug Test	sulfonamide and disulfone drugs	rapid microbial receptor assay	12 min
Charm II Tetracycline Test	tetracycline drugs	rapid microbial receptor assay	12 min

**Table 6:** Examples of rapid specific tests for the detection of antimicrobial residues in milk.

Test	Manufacturer	Sensitivity	Interpretation Method
MilkSafe^TM^ Fast 2BC	Christian Hansen Holding A/S, Hoersholm, Denmark	beta-lactams including cephalexin	Visually, electronic reader
MilkSafe^TM^ Fast 3BTC	Christian Hansen Holding A/S, Hoersholm, Denmark	beta-lactams including cephalexin and tetracyclines	Visually, electronic reader
MilkSafe^TM^ Fast 3BTS	Christian Hansen Holding A/S, Hoersholm, Denmark	beta-lactams, tetracyclines and sulfonamides	Visually, electronic reader
Charm MRL Beta-lactam and Tetracycline Test for Milk	Charm Sciences, Inc., Lawrence, KS, USA	14 beta-lactams and 3 tetracyclines	Charm EZ system
Charm MRL Beta-lactam 1-Minute Test for Milk	Charm Sciences, Inc., Lawrence, KS, USA	beta-lactams	Charm EZ system
Charm MRL Beta-lactam 3 min Test for Milk	Charm Sciences, Inc., Lawrence, KS, USA	14 common beta-lactams	Charm EZ system
Charm MRL Beta-lactam Test for Milk	Charm Sciences, Inc., Lawrence, KS, USA	14 primary beta-lactams	Charm EZ reader
Charm Neomycin, Streptomycin and Gentamicin Test for milk	Charm Sciences, Inc., Lawrence, KS, USA	neomycin, streptomycin gentamicin, kanamycin, dihydrostreptomycin	Rosa Reader, Charm EZ system
Charm QUAD1 Test for milk	Charm Sciences, Inc., Lawrence, KS, USA	beta-lactams, quinolones, sulfonamides, tetracyclines	Charm EZ system
Charm QUAD2 Test	Charm Sciences, Inc., Lawrence, KS, USA	macrolides	Charm EZ system
Beta-lactams+ Tetracyclines BT Combo Test Kit	Ring Biotechnology Co., Ltd., Beijing, China	beta-lactams, tetracyclines	Visually
CCBTS 5in1 Rapid Test Kit	Ring Biotechnology Co., Ltd., Beijing, China	ceftiofur, cephalexin, beta-lactams, tetracyclines, sulfonamides	Visually
BTSC 4in1 QuaTest	Ring Biotechnology Co., Ltd., Beijing, China	beta-lactams, tetracyclines, streptomycin, chloramphenicol	Visually
QuinoSensor Milk	Unisensor S.A., Seraing, Belgium	quinolones	Visually, ReadSensor
TwinSensor^BT^	Unisensor S.A., Seraing, Belgium	beta-lactams, tetracyclines	Visually, Read Sensor
4 Sensor BSCT	Unisensor S.A., Seraing, Belgium	beta-lactams, tetracyclines, chloramphenicol, streptomycin	Visually, ReadSensor
4 Sensor BSTQ	Unisensor S.A., Seraing, Belgium	beta-lactams, sulfonamides, tetracyclines, quinolones	Visually, ReadSensor
4 Aminosensor Milk	Unisensor S.A., Seraing, Belgium	neomycin b, kanamycin a streptomycin, gentamycin	Visually, ReadSensor
BetaXpress	Unisensor S.A., Seraing, Belgium	beta-lactams	Visually, ReadSensor
CapSensor Milk	Unisensor S.A., Seraing, Belgium	chloramphenicol	Visually, ReadSensor
Betastar^®^ 4D Rapid Test	Neogen Corporation, Lansing, USA	beta-lactams, tetracyclines, chloramphenicol, streptomycin	Reader Accusan Pro

**Table 7:** Limits of detection of selected LFIA tests [µg/kg] to antimicrobials [112,113,114,115].

Antimicrobial Agents	TwinSensor^BT^ KIT020	Charm MRLBLTET Test (8 min Test)	BetaStar^®^ S Combo 50	Milksafe^TM^ Fast 3 BTC	MRL (EU)
Penicillin G	2–3	2–3	1.5	1	4
Ampicillin	3–4	2.5–4.0	3	3	4
Amoxicillin	3–4	2.5–4.0	2	3	4
Oxacilin	12–18	-	6	7	30
Cloxacillin	6–8	25–35	5	6	30
Dicloxacillin	6–8	20–30	4	5	30
Nafcillin	30–50	-	20	30	30
Cephapirin	6–8	4–8	20	18	60
Cephalonium	3–5	3–5	2	1	20
Cefazolin	18–22	8–16	90	100	50
Ceftiofur and metabolite	10–15	10–20	30	35	100
Cefoperazone	3–4	3–5	3	2	50
Cefalexin	>750	15–30	3000	18	100
Cefacetril	30–40	6–12	60	60	125
Cefquinome	20–30	20–30	16	16	20
Tetracycline	80–100	10–30	45	35	100
Chlortetracycline	30–40	50–100	80	70	100
Doxycycline	10–15	-	50	30	0
Oxytetracycline	50–60	50–100	50	25	100

**Table 8:** MRLs valid in the EU and according to the Codex Alimentarius [120,121].

Antibiotics	EU MRL [µg/kg]	Codex MRL [µg/kg]
Amoxicilin	4	4
Ampicillin	4	***-***
Benzylpenicillin	4	4
Ceftiofur	100	100
Chlortetracycline, Oxytetracycline, Tetracycline	100	100
Colistin	50	50
Dihydrostreptomycin/Streptomycin	200	200
Danofloxacin	30	-
Erythromycin	40	-
Flumequin	50	-
Lincomycin	150	150
Monensin	2	2
Neomycin	1500	1500
Pirlimycin	100	100
Spectinomycin	200	200
Spiramycin	200	200
Sulfadimidine	100	25
Tylosin	50	100
Gentamicin	100	200

## Data Availability

Not applicable.

## Abbreviations

ADI	Acceptable Daily Intake
AOAC	International Association of Official Analytical Collaboration International
BRT	Brilliant Black Reduction Test
CFU	Colony Forming Units
CRL	The Community Reference Laboratory
EEC	European Economic Community
EGTA	Ethyleneglycol-bis-(b-amino-ethyl ether)-N,N,N,N-tetra-acetic acid
EU-RL	The European Union Reference Laboratories
FAO	Food and Agriculture Organization of the United Nations
FDA	Food and Drug Administration
HACCP	Hazard Analysis and Critical Control Points system
hCG	human chorionic gonadotropin
IDF	International Dairy Federation
ISO	International Organization for Standardization
IU	International Unit
LOD	Limit of Detection
LFIA	Lateral Flow Immuno Chromatography
MRL	Maximum Residue Limit
MRLVD	Maximum Limit for Residues of Veterinary Drugs
STAR method	Screening test for antibiotics residues
WHO	World Health Organization
WTO	World Trade Organization
